# Data in support of crystal structures of highly-ordered long-period stacking-ordered phases with 18*R*, 14*H* and 10*H*-type stacking sequences in the Mg–Zn–Y system

**DOI:** 10.1016/j.dib.2015.09.005

**Published:** 2015-09-25

**Authors:** Kyosuke Kishida, Kaito Nagai, Akihide Matsumoto, Haruyuki Inui

**Affiliations:** a Department of Materials Science and Engineering, Kyoto University, Sakyo-ku, Kyoto 606-8501, Japan; b Center for Elements Strategy Initiative for Structural Materials (ESISM), Kyoto University, Sakyo-ku, Kyoto 606-8501, Japan

## Abstract

The crystal structures of highly-ordered Mg–Zn–Y long-period stacking-ordered (LPSO) phases with the 18*R*, 14*H* and 10*H*-type stacking sequences have been investigated by atomic-resolution scanning transmission electron microscopy (STEM) and transmission electron microscopy (Kishida et al., 2015) [1]. This data article provides supporting materials for the crystal structure analysis based on the crystallographic theory of the order–disorder (OD) structure and the crystallographic information obtained through the structural optimization for various simple polytypes of the highly-ordered Mg–Zn–Y LPSO phases with the 18*R*, 14*H* and 10*H*-type stacking sequences by first-principles density functional theory (DFT) calculations.

**Specifications Table**

**Table:** 

Subject area	*Materials Science*
More specific subject area	*Magnesium alloys, crystal structure, order–disorder (OD) structure*
Type of data	*Text, Image (scanning electron microscopy (SEM)), transmission electron microscopy (TEM)), Table, Crystallographic information in cif format*
How data was acquired	*Scanning electron microscope (JEOL JSM-*7001*FA), Transmission electron microscope (JEOL JEM-*2100F*), WinHREM software package*[3]*and the Vienna Ab initio simulation package (VASP)*[4–6].
Data format	*Raw, Analyzed*
Experimental factors	*High-frequency induction melting in an argon atmosphere*
Experimental features	*Heat-treated at* 500 °C *for* 72 and 300 h*.*
Data source location	*Department of Materials Science and Engineering, Kyoto University, Kyoto, Japan*
Data accessibility	*Data is available with this article.*

**Value of the data**

• The crystallographic data is essential for the investigations of the crystal structures, defect structures and crystallographic orientations of the Mg–Zn–Y LPSO phases by transmission electron microscopy (TEM), scanning transmission electron microscopy (STEM), electron backscatter diffraction (EBSD) in SEM and the other diffraction analysis methods.
• The results of the structural optimization provide useful information for understanding the influences of the stacking relationships between the adjacent structural blocks as well as the additional atoms in Zn_6_Y_8_ atomic clusters on the formation energies of the Mg–Zn–Y LPSO/OD phases and also the phase relationships in the Mg–Zn–Y ternary system.
• The results of diffraction analysis in TEM provides useful information about how the crystal structure evolution can be detected in selected area electron diffraction (SAED) patterns.

## 1 SEM observations

**Fig. 1:**
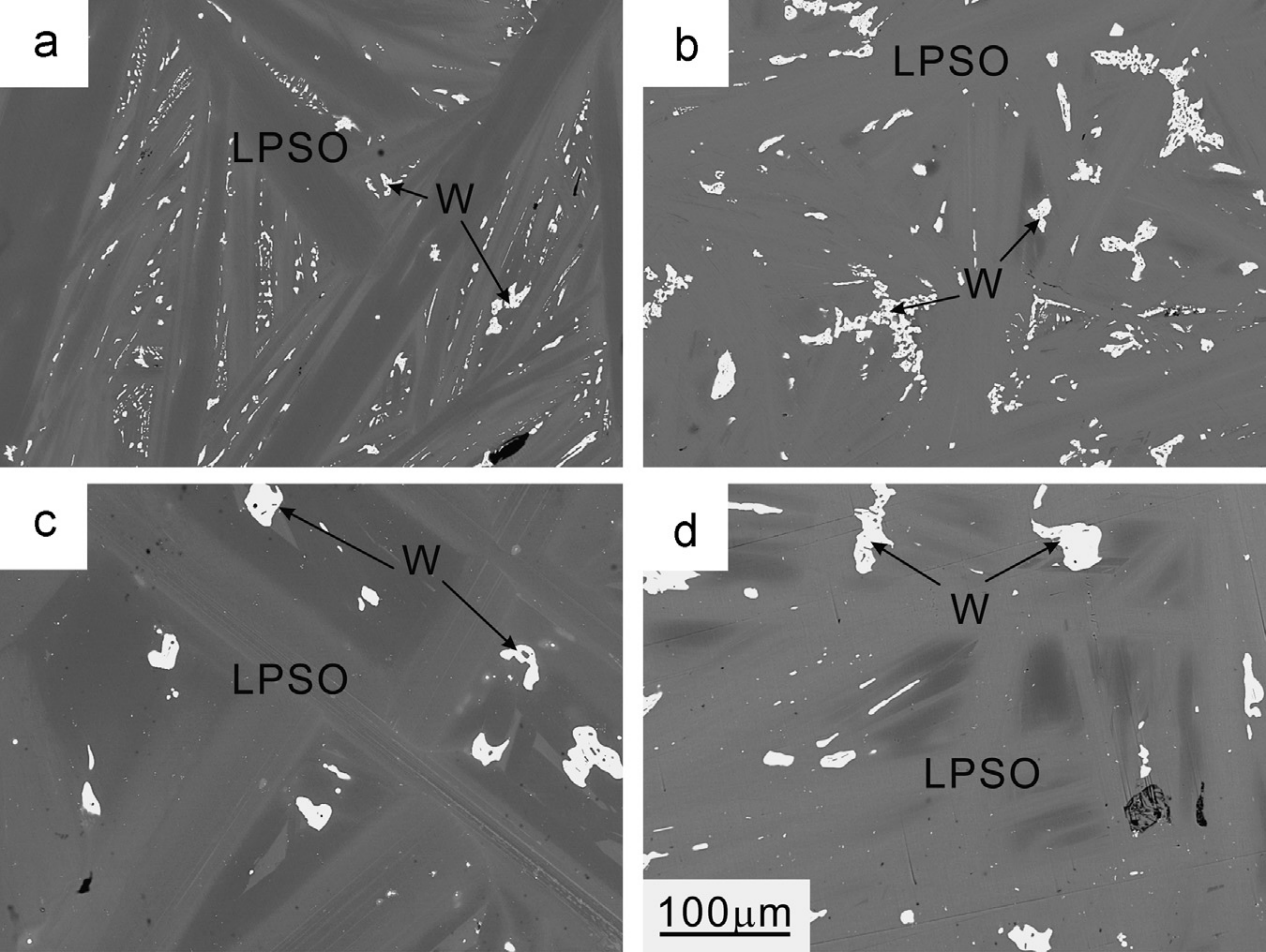
SEM back-scattered electron (BSE) images of alloys (a, c) A and (b, d) B heat-treated at 500 °C for (a, b) 72 h and (c, d) 300 h.

Typical SEM back-scattered electron (BSE) images for alloys A (nominal composition: Mg – 8.3 at% Zn – 11.1 at% Y) and B (nominal composition: Mg – 10.0 at% Zn – 13.3 at% Y) after the heat treatment at 500 °C for 72 h are shown in Fig. 1(a) and (b), respectively. Although the nominal compositions were chosen to obtain single-phase ingots of the fully ordered phases with 18*R*- and 10*H*-LPSO phases, both ingots contain Zn,Y-rich precipitates, which are imaged as the brightest regions in Fig. 1. EDS analysis in the SEM has indicated that the overall compositions for alloys A and B are deviated slightly to the Mg-poor compositions of Mg – 9.2±0.3 at% Zn – 11.2±0.3 at% Y and Mg – 11.4±0.2 at% Zn – 12.8±0.1 at% Y, respectively. The brightest regions in Fig. 1 possess an approximate chemical compositions of Mg – 47.0±0.6 at% Zn – 26.6±0.2 at% Y, which are inferred to be W phase previously reported [2]. The SEM-BSE images for alloys A and B heat-treated at 500 °C for about 300 h indicate that major regions for both alloys still exhibit compositional heterogeneity, which could not be eliminated even after the prolonged heat treatment at 500 °C (Fig. 1(c) and (d)).

## 2 Selected area electron diffraction (SAED) patterns of highly-ordered Mg–Zn–Y LPSO/OD phases

**Fig. 2:**
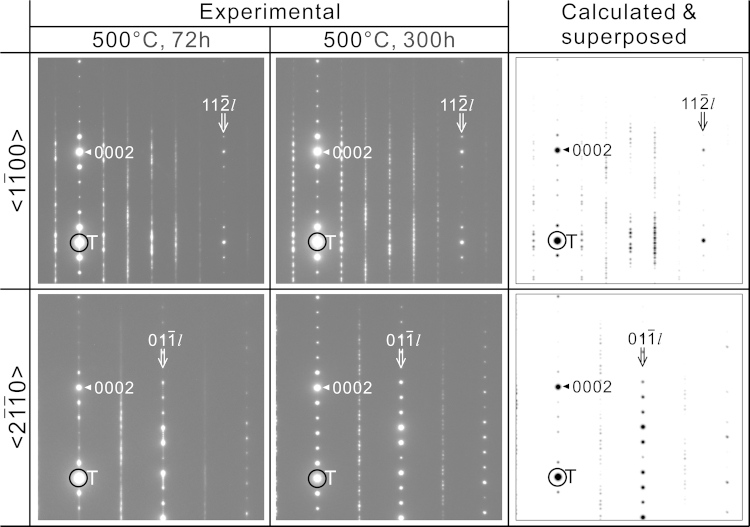
Experimental and calculated SAED patterns for the C_3_-MDO_2_ polytype of the 18 *R*-type Mg–Zn–Y LPSO/OD phase in alloy B heat-treated at 500 °C for 72 and 300 h.

The crystal structure transformation from one-dimensionally disordered structure into the MDO_2_ polytype (2*M*_1_, space group: *C*2/*c* (#15)) belonging to the OD groupoid family of the C_3_-type has been confirmed for the Mg–Zn–Y LPSO/OD phase with the 18*R*-type stacking sequence through the analysis of the atomic resolution STEM images [1]. Fig. 2 shows the corresponding experimental SAED patterns of the $<1\overset{¯}{1}00>$ and $<2\overset{¯}{1}\;\overset{¯}{1}0>$incidences taken from the 18*R*-type Mg–Zn–Y LPSO/OD phase heat-treated at 500 °C for 72 and 300 h. For simplicity, indices to express directions and planes for the LPSO phases are referred to as those of the parental Mg phase with the hcp structure unless otherwise noted. Each of the SAED patterns was obtained from a circular area with an approximately 120 nm in diameter in a grain of 18R-type Mg–Zn–Y LPSO/OD phase. In the SAED pattern taken from the LPSO/OD phase heat-treated at 500 °C for 72 h, the sharp diffraction spots in the reciprocal lattice rows of *n*/6$<11\overset{¯}{2}/>$^⁎^ (*n*=0 and 6) and *n*/2$<01\overset{¯}{1}/>$^⁎^ (*n*=even integers) and intense streaks in the reciprocal lattice rows of *n*/6$<11\overset{¯}{2}/>$^⁎^ (*n*=1, 2, 3, 4 and 5) and *n*/2$<01\overset{¯}{1}/>$^⁎^ (*n*=odd integers) are observed to be coexisted, which is one of the characteristics of the OD structure with one dimensional stacking disorder and can be used as a guide for distinguishing the OD structure with the other crystal structure types, i.e., LPSO and fully-ordered structures by the diffraction analysis in TEM [1]. In contrast, only sharp diffraction spots are observed after the heat-treatment at 500 °C for 300 h, reflecting the crystal structure transformation into a fully ordered structure. The SAED patterns taken from the specimens heat-treated for 300 h are compared with those calculated using the crystallographic parameters given in [1, Table 3] (CIF: 18R-stable_C3-2M1.cif in Supplementary material in this paper) with the WinHREM software package (Fig. 2) [3]. Since the domain structure composed of three differently oriented domains is formed in the 18R-type Mg–Zn–Y LPSO/OD phase [1], the experimental SAED patterns of the $<1\overset{¯}{1}00>$ and $<2\overset{¯}{1}\overset{¯}{1}0>$ incidences coincide well with those obtained after superposing the three SAED patterns calculated for the three different incidences, namely those of ${\left[100\right]}_{2M_{1}}-{\left[\bar{1}10\right]}_{2M_{1}}-{\left[\bar{1}\;\bar{1}0\right]}_{2M_{1}}$ and ${\left[010\right]}_{2M_{1}}-{\left[310\right]}_{2M_{1}}-{\left[\bar{3}10\right]}_{2M_{1}}$, respectively, where the indices with the subscript 2*M*_1_ are referred to the MDO_2_ (2*M*_1_) monoclinic cell.

## 3 First-principles DFT calculations

Structural optimization for various simple polytypes are conducted using the Vienna Ab-initio Simulation Package (VASP) [4]. These simple polytypes are derived based on the crystallographic theory of the order–disorder (OD) structure and are designated as those with the maximum degree of order (MDO polytypes) [1]. The Perdew–Burke–Ernzerhof (PBE) gradient approximation (GGA-PBE) is utilized to treat the exchange-correlation functional [5]. An energy cutoff is set to be 400 eV and Monkhorst-Pack k-point meshes of 6×4×4 (for 1*M* cells), 6×4×2 (for 2*M*, 2*O* and 1*A* calculated with double-sized C-centered cells) and a gamma-centered k-point mesh of 6×6×2 (for 2*H*-type hexagonal cells) are used throughout the calculations [6]. The geometric optimization is terminated when the residual forces become less than 0.01 eV/Å.

Table 1 summarizes the formation energy Δ*E_form_* and stability factor Δ*E_stab_* defined by Saal and Wolverton [7] as well as lattice constants obtained by the first-principle calculations. The Δ*E*_form_ and Δ*E*_stab_ values are evaluated according to the following equations:

(1) $$\Delta E_{\mathrm{form}}=E_{tot}({\mathrm{Mg}}_{l}{\mathrm{Zn}}_{m}Y_{n})-\frac{lE_{tot}(\mathrm{Mg})+mE_{tot}(\mathrm{Zn})+nE_{tot}(Y)}{l+m+n},$$

(2) $$\Delta E_{stab}({\mathrm{Mg}}_{l}{\mathrm{Zn}}_{m}Y_{n})=E_{tot}({\mathrm{Mg}}_{l}{\mathrm{Zn}}_{m}Y_{n})-E_{CH}({\mathrm{Mg}}_{l}{\mathrm{Zn}}_{m}Y_{n}),$$

where *E_tot_*(*i*) is the total energy per atom of the structure (*i*) and *E_CH_*(Mg_*l*_Zn_*m*_Y_*n*_) is the energy of the convex hull at the composition of the LPSO phase. We assume that the convex hull is composed of Mg, MgZnY and Mg_3_Y as proposed by Saal and Wolverton [7].

The crystallographic information files (CIF) for the optimized MDO polytypes of the 18*R*-, 14*H*- and 10*H*-type Mg–Zn–Y LPSO/OD phases with or without an additional atoms at the central site of each Zn_6_Y_8_ atomic cluster are provided as the supplementary materials.

## Figures and Tables

**Table 1:** Formation energy Δ*E_form_*, stability factor Δ*E_stab_* and lattice parameters for some MDO polytypes of Mg–Zn–Y LPSO/OD phases. *c*_SB_ correspond to the height of the structural block.

Stacking sequence type	Stacking relation	MDO polytypes	Space group	Additional atom	Formation energy, Δ*E*_form_ (meV/atom)	Stability factor, Δ*E*_stab_ (meV/atom)	Nearest out-of-plane inter-cluster distance (Å)	Lattice parameters						*c*_SB_ (Å)
*a* (Å)	*b* (Å)	*c* (Å)	*α* (°)	*β* (°)	*γ* (°)
10 *H*	A_1_	2*H*	*P*6_3_/*mcm*	–	−81.9	18.7	13.04	11.21	–	26.08	90	90	120	13.04
Mg	−110.9	−12.0	13.07	11.20	–	26.13	90	90	120	13.07
Zn	−105.8	1.7	13.05	11.18	–	26.10	90	90	120	13.05
Y	−111.0	−6.1	13.07	11.23	–	26.14	90	90	120	13.07
A_2_	2*H*	*P*6_3_22	–	−82.7	17.9	14.56	11.20	–	26.10	90	90	120	13.05
Mg	−111.9	−13.0	14.58	11.20	–	26.13	90	90	120	13.07
Zn	−107.0	0.5	14.55	11.18	–	26.08	90	90	120	13.04
Y	−111.2	−6.1	14.61	11.23	–	26.17	90	90	120	13.09
A_3_	2*O*	*Cmce*	–	−83.2	17.4	14.23	19.40	11.18	26.16	90	90	90	13.08
Mg	−112.3	−13.3	14.22	19.38	11.20	26.14	90	90	90	13.07
Zn	−107.3	0.1	14.20	19.35	11.18	26.11	90	90	90	13.05
Y	−111.1	−6.2	14.25	19.43	11.23	26.19	90	90	90	13.10
A_4_	2*O*	*C*222_1_	–	−82.7	17.9	13.48	11.18	19.37	26.18	90	90	90	13.09
Mg	−111.7	−12.8	13.47	11.19	19.38	26.16	90	90	90	13.08
Zn	−106.8	0.6	13.46	11.17	19.35	26.13	90	90	90	13.07
Y	−111.0	−6.1	13.49	11.22	19.44	26.19	90	90	90	13.10
18 *R*	C_1_	1*M*	*C*2/*m*	–	−99.8	−16.0	16.09	11.16	19.36	16.09	90	103.54	90	15.65
Mg	−124.7	−42.1	16.08	11.16	19.37	16.08	90	103.59	90	15.63
Zn	−120.1	−30.3	16.07	11.15	19.34	16.07	90	103.53	90	15.62
Y	−123.5	−35.8	16.12	11.19	19.40	16.12	90	103.59	90	15.67
2*M*	*C*2/*c*	–	−99.9	−16.0	16.08	11.17	19.34	31.52	90	96.87	90	15.65
Mg	−124.8	−42.1	16.07	11.17	19.36	31.50	90	96.87	90	15.64
Zn	−120.1	−30.3	16.06	11.15	19.33	31.48	90	96.85	90	15.63
Y	−123.4	−35.8	16.11	11.19	19.39	31.56	90	96.87	90	15.67
C_2_	1*M*	*C*2/*m*	–	−99.7	−15.9	15.74	11.17	19.36	15.74	90	96.87	90	15.62
Mg	−124.2	−41.5	15.74	11.17	19.38	15.74	90	96.91	90	15.62
Zn	−119.5	−29.7	15.73	11.16	19.35	15.73	90	96.88	90	15.61
Y	−123.6	−36.0	15.76	11.20	19.42	15.76	90	96.92	90	15.64
2*M*	*C*2/*c*	–	−99.9	−16.1	15.73	11.17	19.37	31.31	90	93.48	90	15.62
Mg	−124.2	−41.6	15.74	11.17	19.36	31.32	90	93.45	90	15.63
Zn	−119.5	−29.7	15.73	11.16	19.33	31.29	90	93.44	90	15.62
Y	−123.6	−36.0	15.76	11.20	19.41	31.36	90	93.49	90	15.65
C_3_	1*A*	*P*1¯	–	−100.0	−16.2	16.40	11.18	11.16	16.40	93.27	103.2	120.0	15.64
Mg	−124.6	−42.0	16.40	11.18	11.16	16.40	93.32	103.2	120.0	15.64
Zn	−119.9	−30.1	16.41	11.16	11.15	16.41	93.33	103.2	120.0	15.64
Y	−123.6	−35.9	16.42	11.22	11.19	16.42	93.22	103.3	120.0	15.65
2*M*_1_	*C*2/*c*	–	−100.0	−16.2	16.39	11.17	19.37	31.32	90	93.53	90	15.63
Mg	−124.7	−42.0	16.38	11.18	19.37	31.31	90	93.41	90	15.62
Zn	−119.9	−30.1	16.37	11.16	19.34	31.29	90	93.43	90	15.61
Y	−123.5	−35.9	16.41	11.20	19.42	31.36	90	93.45	90	15.65
2*M*_2_	*C*2/*c*	–	−100.0	−16.2	16.39	11.17	19.36	31.48	90	96.84	90	15.63
Mg	−124.6	−41.9	16.38	11.18	19.36	31.46	90	96.81	90	15.62
Zn	−119.9	−30.1	16.37	11.16	19.33	31.45	90	96.83	90	15.61
Y	−123.5	−35.9	16.41	11.21	19.41	31.52	90	96.83	90	15.65
2*M*_3_	*C*2/*c*	–	−100.1	−16.3	16.39	11.17	19.36	31.32	90	93.40	90	15.63
Mg	−124.6	−41.9	16.38	11.17	16.37	31.31	90	93.42	90	15.63
Zn	−119.9	−30.1	16.37	11.16	16.34	31.29	90	93.41	90	15.62
Y	−123.5	−35.9	16.41	11.19	16.42	31.36	90	93.44	90	15.65
14 *H*	A_1_	2*H*	*P*6_3_/*mcm*	–	−61.4	10.4	18.18	11.17	–	36.36	90	90	120	18.18
Mg	−82.5	−11.5	18.20	11.17	–	36.40	90	90	120	18.20
Zn	−78.7	−1.6	18.19	11.15	–	36.38	90	90	120	18.19
Y	−82.3	−7.0	18.22	11.19	–	36.43	90	90	120	18.22
A_2_	2*H*	*P*6_3_22	–	−61.3	10.5	19.29	11.17	–	36.37	90	90	120	18.18
Mg	−82.6	−11.6	19.29	11.17	–	36.36	90	90	120	18.18
Zn	−78.8	−1.7	19.27	11.16	–	36.33	90	90	120	18.17
Y	−81.8	−6.5	19.31	11.19	–	36.40	90	90	120	18.20
A_3_	2*O*	*Cmce*	–	−61.2	10.7	19.03	19.37	11.15	36.40	90	90	90	18.20
Mg	−82.4	−11.4	19.03	19.38	11.15	36.39	90	90	90	18.20
Zn	−78.7	−1.6	19.02	19.35	11.13	36.38	90	90	90	18.19
Y	−81.6	−6.3	19.06	19.40	11.18	36.45	90	90	90	18.23
A_4_	2*O*	*C*222_1_	–	−61.3	10.6	18.48	11.15	19.37	36.40	90	90	90	18.20
Mg	−82.3	−11.3	18.48	11.16	19.36	36.39	90	90	90	18.20
Zn	−78.5	−1.4	18.47	11.15	19.33	36.37	90	90	90	18.19
Y	−81.8	−6.5	18.51	11.18	19.40	36.45	90	90	90	18.23

## Footnotes

Appendix A Supplementary data associated with this article can be found in the online version at 10.1016/j.dib.2015.09.005.
